# Adjuvant-independent airway sensitization and infection mouse models leading to allergic asthma

**DOI:** 10.3389/falgy.2024.1423938

**Published:** 2024-08-02

**Authors:** Mariem Radhouani, Philipp Starkl

**Affiliations:** ^1^ Research Division of Infection Biology, Department of Medicine I, Medical University of Vienna, Vienna, Austria; ^2^ CeMM Research Center for Molecular Medicine of the Austrian Academy of Sciences, Vienna, Austria

**Keywords:** allergic asthma, microbial infection, adjuvant-free, natural allergen, mouse model, sensitization

## Abstract

Asthma is a chronic respiratory disease of global importance. Mouse models of allergic asthma have been instrumental in advancing research and novel therapeutic strategies for patients. The application of relevant allergens and physiological routes of exposure in such models has led to valuable insights into the complexities of asthma onset and development as well as key disease mechanisms. Furthermore, environmental microbial exposures and infections have been shown to play a fundamental part in asthma pathogenesis and alter disease outcome. In this review, we delve into physiological mouse models of allergic asthma and explore literature reports on most significant interplays between microbial infections and asthma development with relevance to human disease.

## Introduction

Asthma is the most prevalent chronic respiratory disease with over 260 million cases worldwide (1). This heterogenous lung disorder leads to airway obstruction that can severely affect basic lung function. Asthma is now recognized as a syndrome encompassing a large array of symptoms, such as shortness of breath, wheezing, coughing, as well as chest tightness and pain with fluctuating severity (2–4).

Classically, asthma has been viewed as the archetype of a type 2 immunity-mediated, allergic disease. Many asthmatic patients show enhanced allergen-specific immunoglobulin E (IgE), eosinophilia and a predominant type 2 T helper (Th2) cell phenotype in the blood and lung lavage fluid (5). However, more recent evidence also suggests the existence of non-Th2-driven asthma (6). For the sake of simplicity, we here use the sole term “asthma” referring to the Th2-driven disease endotype.

Successful translational research requires comprehensive understanding of allergic responses in physiologically relevant contexts. On this basis, murine models have emerged as indispensable tools, unraveling intricate immunopathological and molecular mechanisms while serving as platforms for the evaluation of innovative treatment strategies. In the early 1990s, researchers successfully established mouse models that recreated several key features of allergic asthma, such as IgE production, airway remodeling, eosinophilia and bronchial hyperresponsiveness (7). These tools have greatly facilitated our understanding of key immune mechanisms during acute allergic inflammation in context of the entire organism.

However, most of the early asthma models required unnatural sensitization routes (i.e., intraperitoneal injections) and the use of isolated non-respiratory allergens with low immunogenicity, such as chicken ovalbumin (OVA), alongside powerful chemical adjuvants to achieve a potent type 2 immune response (8, 9).

Over the last decades, more and more physiological asthma mouse models have been developed that use, for instance, extracts of naturally occurring allergen sources and routes of exposure that are relevant for humans. These frameworks could recapitulate the key features of type 2 immune responses and, importantly, some of the complex pathological mechanisms of asthma (3, 10).

In this review, we aimed to provide an overview of adjuvant-free murine asthma models and discuss their relevance to human disease. Special emphasis was placed on the application of naturally occuring allergens (as part of allergen extracts or derived purified components in relation to human disease and sensitization). To integrate the context of physiologic environmental modulators of asthma, we discuss important microbes that can evidently initiate or potentiate allergic immune responses and airway inflammation.

## Modeling the key characteristics of allergic asthma

### Hallmarks of allergic asthma

**Figure 1:**
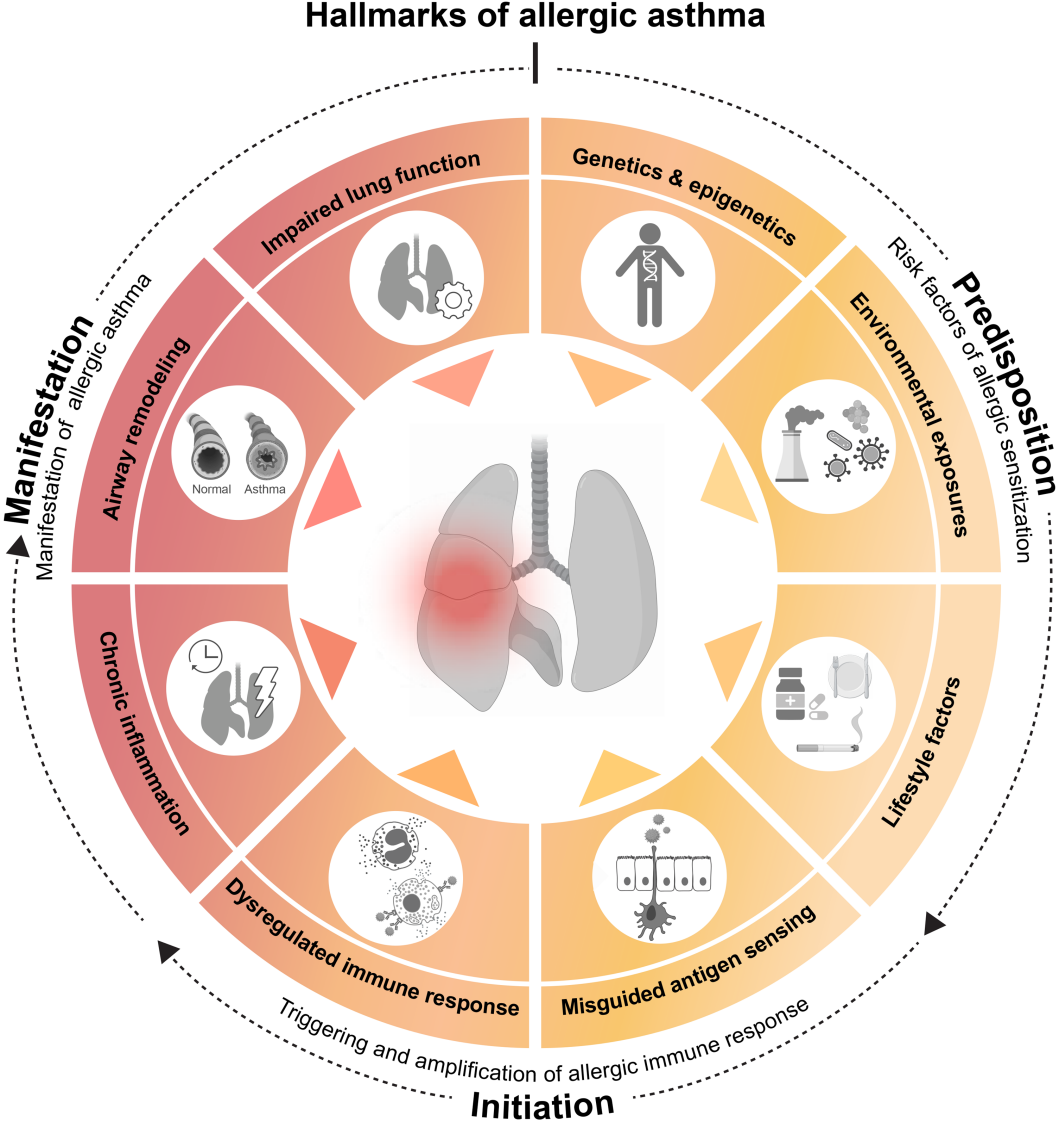
**Hallmarks of allergic asthma**. Asthma is a multifactorial disorder whose initial development seems influenced by predisposing factors, such as (epi)genetics, environmental exposures and lifestyle. The underlying cascade of immune events is often triggered and amplified by a misdirected immune response against apparently harmless environmental substances. Repeated allergen exposure of the airway then results in the clinical manifestations of the disease. On basis of these observations and in an attempt to simplify a highly complex pathology, we propose the following 8 hallmarks of allergic asthma. Predisposing hallmarks which encompass major risk factors [(1) genetic and epigenetic determinants, (2) environmental influences, such as pollution or microbes and (3) lifestyle factors, such as smoking, medication or dietary habits] that facilitate hallmarks of initiation [based on (4) misguided sensing of environmental substances as noxious, leading to (5) a dysregulated immune response], resulting in allergic sensitization. Finally, recurring airway allergen exposure results in manifestation hallmarks of allergic asthma, consist of major clinical and pathological characteristics [(6) chronic lung inflammation, (7) airway remodeling, and (8) impaired lung function]. This illustration was created with BioRender.com.

Asthma is a multifactorial heterogenous disorder. Its development is thought to be influenced by a variety of predisposing factors, such as genetic background, lifestyle and environmental exposures (11) (Figure 1). Asthma is primarily defined by a narrowing of the airways, which is mediated by two main events: initially, an inflammation within the airway lining manifests, characterized by immune cell activation and infiltration and consequent inflammatory mediator production (3, 10). The chronicity of this inflammatory phase will subsequently allow a complex and prolonged interplay between airway immune and structural cells, leading to tissue remodeling. These changes in pulmonary structure are driven by epithelial cell activation, muscle cell hyperplasia, goblet cell metaplasia and hypervascularity. Pathologic airway remodeling typically associates with excessive collagen deposition, mucus hypersecretion or mucus plug formation and thickening of the airway wall, often resulting in lung dysfunction (10, 12).

The immunological features of asthma have been instrumental in segregating the various endotypes of this disorder (5, 7). The most notable immunological milestones from asthma initiation to manifestation include pulmonary infiltration of immune cells [such as dendritic cells (DCs), eosinophils, neutrophils, mast cells, type 2 innate lymphoid cells (ILC2s) and lymphocytes] as well as secretion of type 2 cytokines [e.g., interleukin (IL)-4, IL-5, IL-13] and chemoattractants (e.g., eotaxins and complement anaphylatoxins). The imbalanced Th cell (Th2 dominant) response often leads to allergen-specific IgE and IgG1 production (5). In this review, we refer to these antibodies collectively as type 2 antibodies due to the dependence of their production on type 2 cytokines (IL-4 and IL-13) (13).

### Modeling asthma in mice without adjuvants

**Figure 2:**
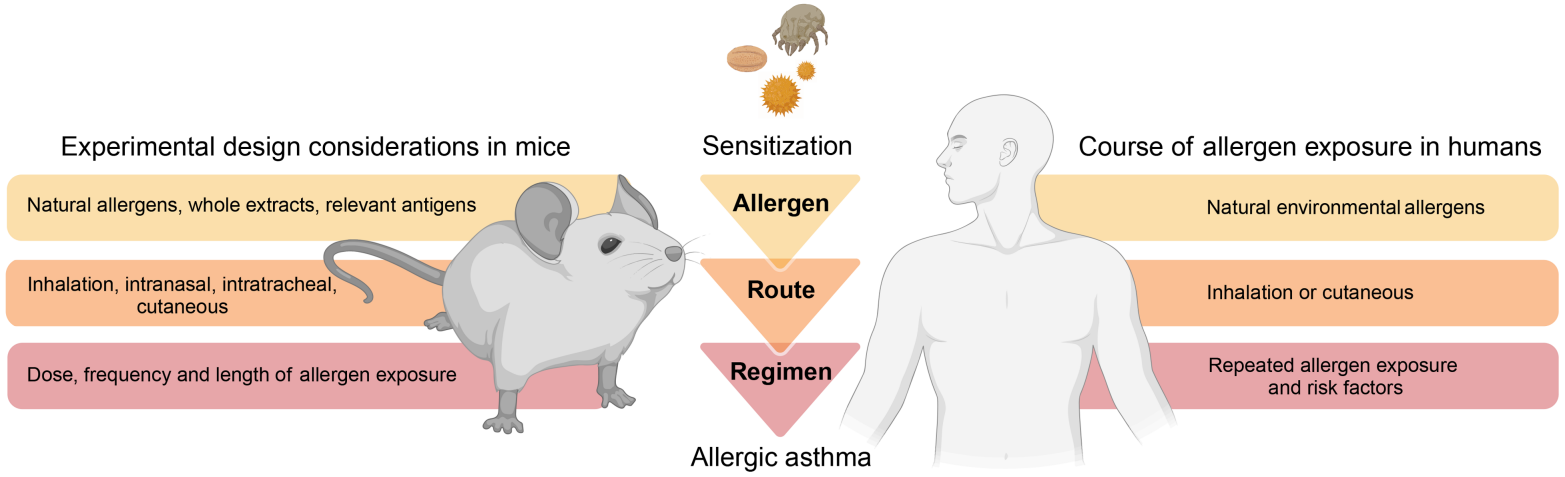
**Modelling allergic asthma: mouse vs human**. Mouse models of allergic asthma response ideally implement fundamental principles that consider physiologic aspects related to disease onset and progression in humans. As such, mouse models should consider physiologic allergen exposure conditions via barrier organs, utilize relevant allergens and avoid clinically irrelevant adjuvants, as well as incorporate realistic protocols of allergen exposure. This illustration was created with BioRender.com.

A considerable difference to humans is that mice housed in a laboratory environment do not naturally develop allergic airway inflammation and hyperresponsiveness but require an artificial, experimental framework for asthma onset (14). To faithfully and realistically replicate an allergic response in mice, most protocols typically involve a two-phase approach (8). The first phase of initial allergen exposure (referred to as “sensitization”) leads to an induction of antigen-specific type 2 immunity. The second phase (referred to as “challenge”) encompasses secondary allergen exposure(s) of the sensitized individual and results in an allergic reaction and inflammation. The sensitization protocols and experimental approaches can vary greatly between studies. However, one could consider that “physiological” mouse models of asthma rely on three fundamental principles to facilitate clinical and pathological translation (Figure 2): (1) physiologic allergen exposure conditions via barrier organs (skin or airways); (2) use of clinically significant allergens (i.e., with reported sensitization in humans) and allergen extracts and avoidance of clinically irrelevant (i.e., no evidence for driving sensitization in humans) adjuvants; (3) appropriate exposure regimen that consider realistic allergen doses as well as exposure frequency and duration to eventually recreate the main features of human asthma.

### Adjuvant-independent mouse models of allergic asthma

Various mouse models based on natural allergens were developed to investigate the mechanisms of allergen-driven airway immune and functional responses during acute and established inflammation (15). In this section, we review and discuss examples of such protocols that recreate main features of allergic asthma in mice using relevant allergens in absence of chemical adjuvants. All mouse models and treatment protocols are summarized in (Table 1).

**Table 1:** Adjuvant-free allergic asthma mouse models using natural allergens.

Allergens	Protocol	Observed allergy features	Presence of airway inflammation and/or hyperresponsiveness	Mice (strain, sex, age)	References
House dust mite (*Dermatophagoides pteronyssinus*) extract	Sensitization: 1 μg or 100 ng HDME i.t. extract; challenge: i.n. 10 μg HDME daily from day 7 to 11;	ILC2s, DCs and eosinophilia; Airway hyperresponsivenes; type 2 dominated lung immune response;	Inflammation and airway hyperresponsiveness	C57BL/6 females, 6–12 week old	(16–19)
House dust mite extract (*Dermatophagoides pteronyssinus*)	Acute model: HDME i.n. sensitization with 100 µg for 5 consecutive days then challenge on day 11 with the same dose; 4-week chronic model: i.n. sensitization with 25 or 10 µg HDME for 5 days then challenge with the same doses 5×/week for 3 weeks; 8-week chronic model: i.n. sensitization with 25 µg HDME for 5 days. 2 days rest then 3×/week for 7 weeks with the same dose;	All models: increased airway hyperresponsiveness to methacholine; acute model: high lung IL-4, IL-17, CXCL1 and IL-6; chronic model: comparable airway remodeling features and similar numbers of immune cells between the 4 and 8-week protocols;	Inflammation and airway hyperresponsiveness	BALB/c females, 8–10 week old	(20)
House dust mite extract (*Dermatophagoides pteronyssinus*)	i.n. sensitization with 10 μg HDME; challenge from day 7 to 11 with 20 μg daily;	Airway hyperresponsiveness; immune cell infiltration; mucus secretion; serum IgE; lung eosinophilia; BAL IL-13 and IL-5;	Inflammation and airway hyperresponsiveness	BALB/c females, 6–8 week old	(21)
Cockroach (Purified serine protease Per a 10)	i.n. 25 μg of Per a 10 at days 0, 2, 4, 6, 10, 12 and 14	Increased lung IL-33, TSLP, IL-1α, uric acid and eosinophil peroxidase Increased total serum IgE	Inflammation	BALB/c females, 6–8 week old	(22)
Cockroach fecal remnants (frass)	i.t. frass 40 µg exposure	Mixed Th2 and Th17 lung immune response	Inflammation	BALB/c and C57Bl/6 females, 6 week old	(23)
Cockroach (whole extract)	i.n. 50 μg of extract 5×/week then challenged at day 11 for 4 consecutive days or until day 23 5×/week	Long term exposure: eosinophilic inflammation, AHR and cockroach specific IgG1 Short-term exposure: increased IL-4, CCL11, CXCL1 and CCL2 cytokines	Inflammation	Male BALB/c mice and C57Bl/6 mice (6–8 week old)	(24)
Grass pollen (allergen Phl p5b)	s.c sensitization with 6 injections of 20 μg recombinant Phl p 5b every 2–3 days. 5 days after the last injection, one s.c. injection of 1 mg allergen. A month later: challenge i.n. with 50 μg allergen 1×/week for 3 weeks	Eosinophil infiltration IL-5 production by ex-vivo stimulated splenocytes Airway hyperresponsiveness	Inflammation and airway hyperresponsiveness	BALB/c females, 5–6 week old	(25)
House dust mite extract (*Dermatophagoides pteronyssinus*)	i.n. of 25 μg HDME for 5 days/week for up to 7 weeks	Early type 2 cytokine production in lung; eosinophil and Th2 cell infiltration; airway resistance;	Inflammation and airway resistance	BALB/c females, 6–8 week old	(26)
House dust mite extract (*Dermatophagoides pteronyssinus*)	15 μg HDME for 3 days/week for 3 weeks	IL-25 mediated allergic response with a type 2 dominant phenotype; Airway remodeling (collagen deposition, smooth muscle hyperplasia and airway hyperreactivity); Serum total and HDM-specific IgE;	Inflammation and airway resistance	BALB/c females, 6–8 week old	(27)
House dust mite extract (*Dermatophagoides pteronyssinus*)	i.n. 50 μg HDME 3×/week for either 4 or 15 weeks; Some experiments were followed by a “rest” period, HDME dose was lowered to 25 μg 3×/week for 9 weeks	Bone marrow, lung and serum IgE, IgG1 and Derp1-specific IgE; IgE ^+^ plasma cells and mast cell degranulation;	not reported	C57BL/6 wild type (WT) and transgenic mice on C57BL/6 background, sex and age not specified	(28)
House dust mite extract (*Dermatophagoides pteronyssinus*)	Whole HDME extract containing the equivalent of 23 μg of Der p 1 protein i.n. for sensitization; Challenge i.n. with equivalent of 5.75 μg of Der p 1 on days 10 to 14	Der p 1^+^ CD4^+^ T cells (Th1) in lung, spleen and lymph nodes; lung eosinophils and glycogen deposition in lung histology;	Inflammation and airway hyperresponsiveness	Male and female C57BL/6 WT and transgenic mice, age not specified	(29)
House dust mite extract (*Dermatophagoides pteronyssinus*) and *Alternaria alternata* extract	i.n. administration of 10 μg HDME on day 0; i.n. challenge on day 7 with 10 μg HDME daily for 5 days; 6 to 12 weeks later, mice were either left untreated or rechallenged with 10 μg HDME i.n. once or 10 μg of *Alternaria alternata* extract	Lung inflammation and mucus hypersecretion; airway hyperresponsiveness; lung eosinophils, type 2 cytokines and chemoattractants; memory Th2 cells in BAL, lung and lymph nodes;	Inflammation and airway hyperresponsiveness	Male and female C57BL/6J (WT) and CD45.1, CD45.2 and transgenic mice, 6–12 week old	(30)
House dust mite extract (*Dermatophagoides pteronyssinus*) and LPS	i.n sensitization with 50 μg HDME daily for 3 days then i.n. challenge (5 μg daily on days 14 to 17; in some experiments, mice received i.n. 50 ng LPS on days 20 and 22; CS responsiveness tested with 1 mg/kg dexamethasone i.p. on days 20 and 22	Lung neutrophils, basophils, ILC2s and memory CD8^+^ T cells; lung type 2 cytokines; lung hyperresponsiveness and mucus hypersecretion; resistance to corticosteroid treatment;	Inflammation and airway hyperresponsiveness	Male and female BALB/c mice, 6–8 week old	(31)

### Acute allergic airway inflammation models

#### House dust mite

House dust mites (HDMs; mainly *Dermatophagoides pteronyssinus* and *Dermatophagoides farinae*) are among the most prevalent sources of indoor allergens and widely used to replicate allergic asthma in mice (15, 32). HDMs produce diverse allergenic proteins, including the major allergens Der p 1 and Der p 2, most of which are molecularly and functionally characterized (33–35). Additionally, HDMs are known carriers of various bacteria, fungi and pollutants (36, 37). In combination with HDM allergens, these environmental components are potent triggers of the innate and adaptive immune system, thus driving the allergic sensitization to HDMs. Several research groups have demonstrated allergic sensitization against HDMs using a model of intratracheal (i.t.) sensitization with HDM-extract (HDME), followed by intranasal (i.n.) allergen challenges (16–19). Most protocols consisted of one i.t. low dose (1 µg) HDME instillation at day 0, followed by 10-fold higher i.n. allergen doses 6 or 7 days later for 5 consecutive days and endpoint analyses approximately 3 days after the last challenge (16–19) (Table 1). Such treatments induced a robust Th2 immune response, driven by epithelial cell damage and activation of DCs, granulocytes (mast cells, eosinophils, basophils) and ILC2s (16–19). Additionally, most of these studies reported bronchial hyperreactivity, restructured lung architecture and mucus secretion as well as significant HDM-specific IgE production (17, 19). Other protocols rely on i.n. administration of HDME for both, allergen sensitization and challenge (20, 21, 26, 27) with the advantages that i.n. requires less experimental training and equipment than i.t. application while being similarly relevant for respiratory allergen exposure. Compared to models utilizing also i.t. application, these exclusively i.n.-based models use increased allergen doses (10–100 μg) or a higher frequency and longer exposure periods during the sensitization and challenge phases (20, 21, 26, 27) (Table 1). These protocols recreated the main features of asthma, including pulmonary type 2 cytokine secretion and leukocyte infiltration, lung inflammation, impaired lung function and increased IgE and IgG1. In summary, both, i.n. and i.t. HDME instillations reproduce robust type 2 immune-dominated inflammation in the lung with comparable pathology and functional impairment.

Despite standardized production protocols and environments, significant batch-to-batch and lot-to-lot variation in commercial HDME has been observed, reflected by differences in protease activity as well as allergen, lipopolysaccharide (LPS), chitin and ß-glucan content. These variations could be attributed to differential HDM culture conditions. Indeed, several studies have demonstrated that the composition of culture media, growth conditions of HDMs and their diet can alter the production of relevant allergens (38–41).

For instance, two recent studies have compared different HDME lots and their potency to induce allergic immune responses (42, 43). Assessing six HDME lots differing in total protein, antigen, and endotoxin contents, Cyphert-Daly et al. (42) reported that Der p 1 concentration in the HDME correlates with lung IL-5 and eosinophilia, serum IgE as well total protein in bronchoalveolar lavage (BAL) fluid. While only some of the extracts were able to induce airway resistance, all lots induced peribronchial inflammation and increased mucus secretion. Notably, the lots were tested in two different HDM allergy models: an acute asthma model with weekly i.n. HDME instillation for 3 weeks and a setting reflecting chronic sensitization based on one intraperitoneal (i.p.) HDME injection, followed by weekly oropharyngeal allergen administration from week 3 to 7 post sensitization (42). The second study by Pascoe et al. (43) compared two lots of HDME with either low or high LPS levels but similar in Der p 1 abundance. The mice were treated i.n. with 25 μg HDME 5 times a week for two weeks. The authors found that LPS differences did not influence HDME-induced pulmonary immune cell infiltration or IL-4 in the BAL fluid. However, LPS-high HDME resulted in increased transforming growth factor ß (TGFß) and decreased IL-5 (compared to LPS-low HDME) and the exclusive induction (compared to mock treatment) of Eotaxin, IL-17 and RANTES. Remarkably, only LPS-low HDME induced significant vascular endothelial growth factor (VEGF) levels in BAL fluid. In addition, HDME application induced a broad lung transcriptional response that was specifically influenced by LPS levels. Another important observation of the study was that LPS-high HDME appeared to impair lung function more efficiently.

Addressing a related question, Hadebe et al. compared the effects of LPS and ß-glycan on HDME-mediated airway inflammation (44) using a model used was based on two i.t. applications of HDME alone or in combination with LPS and ß-glucan, followed two weeks later by challenges on three consecutive days. Presence of LPS and ß-glycan resulted in significantly higher pulmonary neutrophil infiltration as well as IL-17, CC chemokine ligand (CCL)3 and CCL5. However, HDME alone caused more mucus production, airway resistance and lung tissue cytokines (IL-4, IL-5 and IL-6) (45).

It should be emphasized that most of the referenced studies used female mice due to their enhanced susceptibility for allergy development (46). Additionally, BALB/c mice were mostly the strain of choice. This detail is relevant since genetic mouse strain differences can influence the outcome of type 2 immune responses, for instance against parasites (47, 48). However, a study by HayGlass and colleagues has reported no inherent Th1/Th2 bias in allergen-driven responses in mice immunized with recombinant (r)Der p 1, OVA or human serum albumin with adjuvant in BALB/c vs. C57BL/6J mice (49). More recently, the key role of the MHC II haplotype for allergic sensitization has been reported (50). As such, the H2d MHC II haplotype of BALB/c mice is associated with an inefficient antibody response to Der p 2 while the H2b MHC II haplotype of C57BL/6 mice seems to drive the strong humoral response against the allergen observed in this mouse strain (50). Additionally, these two strains showed differences in the pulmonary cellular response to Der p 2, with a predominantly eosinophilic phenotype in C57BL/6J vs. neutrophilic in BALB/c mice (50). This important finding suggests that the immune response to whole HDM extracts in BALB/c mice is predominantly attributed to Der p 1 (and allergens other than Der p 2) and potentially other environmental extract constituents. Importantly, genetic background has also been tied to inherent structural and physiological differences in the lungs of BALB/c vs. C57BL/6J mice (51–55). For instance, C57BL/6J mice display a greater lung elastance as well as lung collagen content than BALB/c (51–54). Moreover, strain-dependent differences were also reflected by differing eosinophil distribution and localization within the lung (53).

Another important point to consider in context with allergen exposure in mouse models is the dose relevance. Several studies investigated personal indoor mite exposure over time (56–61). The devices used to sample aeroallergens in the environment varied from portable air samplers to intranasal or body-worn samplers (56–61). Based on these investigations, the mean aeroallergen concentration (presented in Der p 1, allergen or protein units) during undisturbed conditions ranged from 0 to 1.7 ng allergen/m^3^. During human activities (e.g., vacuuming, changing bedsheets, or outdoor transport), it ranged from 0.3 to 190 ng allergen/m^3^ (56). While most these studies focused on the mean allergen levels as opposed to other statistical measures, such as peak allergen concentrations (56), Swanson and colleagues reported a peak allergen exposure of 736 ng house dust mite allergen/m3 after 5 min of making a bed (61). This is substantially lower than the doses of HDME typically used intranasally in mice which range from 1 to 100 µg (Table 1). Thus, additional mouse models relying on lower HDME doses (but perhaps at a higher frequency) during sensitization and challenge could model human exposure and sensitization in a more relevant way.

Overall, these findings corroborate the relevance of HDME-mediated asthma mouse models to study allergic immune responses and various human disease endotypes (5, 10). A special emphasis must be put on the composition of HDME as it can affect several aspects of the allergic asthma response. In this context, it is advisable to use the same or comparable extract batches throughout a study. In addition, we encourage authors of experimental studies to disclose available details on used extracts, such as batch identifiers as well as allergen or LPS content, in the methods sections of their research papers.

#### Cockroach

Cockroaches represent another important environmental source of natural allergens with high relevance to human asthma (62). Indeed, cockroach allergens are frequently detected in urban homes in the United States and can trigger positive skin prick tests in the majority of asthmatic inner-city children (62, 63). Several cockroach components have been identified as potent IgE-inducing allergens, mainly in the German (*Blatella germanica*; Bla g allergen groups) and American cockroaches (*Periplaneta americana*; Per a allergen groups) (62). Many cockroach-mediated asthma mouse models involve the use of adjuvants (64–67). However, several studies have reported effective adjuvant-free protocols based on i.n. sensitization to cockroach allergen (Per a 10) (22) or whole extract (24), or i.t administration of cockroach fecal remnants (23). These models (Table 1) were associated with increased airway inflammation and resistance, enhanced cytokines [thymic stromal lymphopoietin (TSLP), IL-13, IL-4, IL-5 and Eotaxin] in lung tissue or bronchoalveolar lavage (22–24), and type 2 (IgE (22) and IgG1 (24)) antibody production.

#### Grass pollen

Pollen allergy is amongst the most important risk factors for asthma onset and exacerbations (68, 69). In patients with asthma and seasonal allergic rhinitis, outdoor pollen exposure can efficiently induce type 2 immune responses, including eosinophilia, type 2 cytokines and IgE antibodies (69, 70).

Most of the currently applied pollen allergy and asthma mouse models rely on i.p. sensitization of allergen extracts mixed with potent adjuvants, such as Alum (71–73). In a recent study, Stiehm and Peters used the major grass pollen allergen Phl p 5b without adjuvant (Table 1). The model was based on six subcutaneous injections every 2–3 days for the sensitization phase, followed by 3 i.n. treatments with 1 week-intervals. The treated mice showed high levels of eosinophils, lymphocytes and IL-5 in the BAL fluid, increased lung inflammation and type 2 antibodies. Allergic animals also exhibited increased airway resistance to methacholine challenge (25).

### Chronic allergic airway inflammation and severe asthma models

Asthma is importantly defined by its chronicity and several research groups have strived to recapitulate this aspect in mouse models (9). However, modeling of chronic asthma is notoriously difficult as mice usually develop tolerance to the applied allergen over time and possess considerable disease-relevant physiological and anatomical differences as compared to humans (74, 75). Mouse models were nevertheless used to recapitulate some of the airway remodeling features observed in human asthmatics. Typically, these models require a lengthy and frequent allergen exposure of 7–10 weeks (20, 76, 77). Two of the protocols utilized i.n. HDME treatments with 5 allergen applications per week, resulting a profound lung type 2 cytokine milieu, airway remodeling with eosinophilic inflammation, and increased allergen-specific antibodies. Woo et al., compared a 4-week allergen exposure to the classical 8-week model and found that the lung function and airway remodeling parameters were comparable between the two groups. Sibilano and colleagues used a an 11-week model based on 3 initial i.n. (100 µg) HDME instillations for sensitization in the first week, followed by 10 weekly i.n. applications (20 µg) to maintain the immune response and model chronic exposure (77).

An elegant study by Asrat and colleagues investigated IgE production in short vs. long-term HDME exposure (28). The protocol relied on the i.n. administration of 50 μg HDME 3×/week for 4 or 15 consecutive weeks followed or not by a “resting” period with lowered (25 μg) HDME dose (28). The long-term allergen exposure led to the production of long-lived IgE^+^ plasma cells that accumulate in the bone marrow and were able to produce anaphylactic IgE. Importantly, these bone marrow IgE^+^ plasma cells were also found in humans and allergen-specific IgE produced by these cells effectively induced mast cell degranulation following transfer into mice (expressing a partially humanized high affinity IgE receptor FcɛRI) and HDME treatment. In contrast, the short-term HDM exposure led to IgE^+^ plasma cells that reside in secondary lymphoid organs and recipient mice engrafted with IgE-containing serum did not respond with mast cell degranulation upon allergen challenge (28). These findings highlight the great translational relevance of HDME-driven allergic asthma mouse models in the context of serological memory and IgE responses. In line with memory immune responses to HDM allergens, Hondowicz et al. used a shorter term HDME exposure model to characterize the formation dynamics of Der p 1-specific tissue resident (Trm) and circulating memory (Tcm) Th2 cells and to show their dependence on IL-2 for migration and residence (29). A more recent study by Rahimi et al. applied a long term HDME exposure combined with HDME or *Alternaria alternata* challenge (Table 1) to highlight the transcriptional and functional differences between Trm and Tcm Th2 cells (30). While Trm Th2 cells drove airway remodeling features including mucus hypersecretion and airway hyperresponsiveness, Tcm Th2 cells induced eosinophil and CD4^+^ T cell accumulation in the lung (30). These studies provide an important insight into the establishment and function of allergen-specific memory type 2 immune responses and underline the human relevance of these HDME-based models.

Aside from chronicity, severe asthma and asthma exacerbations often exhibit a signature resistance to standard corticosteroid (CS) treatment (78, 79). Resistance to CS is attributable to various mechanisms leading to altered glucocorticoid signaling at different stages (78). CS treatment is known to induce IL-10 production in asthmatics and therefore dampen inflammation. However, this response is dysfunctional or insufficient in patients with severe asthma (80). Additionally, steroid responsiveness seems to be highly dependent on the inflammatory profile present in the lung (80, 81). For instance, many severe asthmatics show an elevated IL-17 signature (82–84) which plays a critical role in Th17/neutrophilic steroid-resistant asthma (85). Numerous mouse models have been used to show features of refractory asthma with most protocols relying on recombinant cytokines, immune cell transfers or allergenic and pathogenic exposures. Key molecules such as LPS, interferon (IFN)*γ* were linked to the development steroid-resistant asthma (81, 86, 87). Gauthier and colleagues suggested a CXCL10 signature in human and mouse Th1-driven severe asthma (88). Using single cell RNA-sequencing, a more recent study has provided deeper insights into the immune landscape of CS-resistant asthma (31). This work used an HDME-based mouse model followed by LPS challenge (Table 1) and identified basophils, ILC2s and CD8^+^ memory T cells as primary producers of IL-4 and IL-13 (31). Moreover, inhibition of IL-13 efficiently reduced HDME/LPS-induced airway hyperresponsiveness and inflammation and could be used to bypass CS-resistance (31). Overall, it appears that balanced T cell responses and certain innate immune mediators are crucial for steroid responsiveness.

While murine asthma models still have limitations in reproducing chronic allergen exposure and airway remodeling features, they represent valuable tools to study the immune responses and molecular pathways driving onset and development of allergic asthma.

## Environmental pathogens that potentiate allergic asthma

Commensal and pathogenic microorganisms actively interact with our immune system and modulate its specificity, sensitivity and maturity as well as the amplitude of immune responses (89–91). Environmental microbes are therefore prime candidates as modulators of allergy development. As discussed in the previous section, several pathogen constituents, such as bacterial or fungal materials (e.g., LPS and ß-glucan), are potent contributors to asthma induction and exacerbation. Most allergen extracts, for instance those derived from HDM, cockroach and pollen and even OVA preparations, contain variable, but often substantial amounts of LPS which can potentially shape immune responses against allergens (92). Both, the lung and gut microbiota seem to significantly modulate and exacerbate allergic asthma (90, 93–95). In this section, we focus on the current evidence of pathogenic microbes and infections as modulators of asthma development in experimental murine studies. In our search, we considered clinically relevant respiratory pathogens (96–98) and summarize study examples in Tables 2–4.

**Table 2:** Experimental studies testing the effects of viral infections on allergic asthma.

Viruses	Protocol	Observed allergy features	Presence of airway inflammation and/or hyperresponsiveness	Mice (strain, sex, age)	References
Human rhinovirus (HRV-1B)	i.n. inoculation with 10^8^ TCID_50_ HRV-1B or inactivated virus; 2 days later, i.n. 25 μg of HDM protein (equivalent of 144 μg of whole-crushed HDME) for 10 days	Lung neutrophils and macrophages; serum HDME-specific IgG1 and total IgE; airway hyperresponsiveness;	Inflammation and airway hyperresponsiveness	BALB/c females, 7 week old	(99)
Human rhinovirus (HRV-1B) exacerbation	i.n. sensitisation for 3 days with 50 μg HDME, then daily challenge with 5 μg HDME on days 14 to 17; on day 18, mice were i.n. infected 5 × 10^6^ virions (TCID_50_) or UV-inactivated virus; Girkin et al. used 10^7^ virions for the HRV-1B infection	BAL and lung lymphocytes and eosinophils; interferons, type 2 cytokines and alarmins; increased airway resistance and hyperresponsiveness;	Inflammation and airway hyperresponsiveness	BALB/c WT and transgenic male and females mice, 6–14 week old	(100–102)
Human rhinovirus (HRV-1B) exacerbation	i.t. 100 μg HDME. 10 days later, i.n. with 10 μg for 2 days; 24 h later, i.n. inoculation with 2.5 × 10^6^ TCID50/ml HRV-1B or inactivated virus	Lung eosinophils and lymphocytes; total IgE and NETs; airway resistance and mucus hypersecretion;	Inflammation and airway hyperresponsiveness	BALB/c WT males and females, 6–8 week old	(103)
Human rhinovirus (HRV-C15 and HRV-A1B)	i.n. sensitization with 100 μg HDME and challenge with 10 μg on days 11 and 12; on day 13, mice were i.n. inoculated with 5 × 10^6^ PFU equivalents (ePFU) of HRV-C15, HRV-A1B or sham	Lung neutrophils, monocytes, lymphocytes, eosinophils and ILC2s; interferons, type 2 cytokines, chemokines and alarmins; airway hyperresponsiveness;	Inflammation and airway hyperresponsiveness	BALB/c females, 8–12 week old	(104)
Rhinovirus (dsRNA:RV-mimic)	i.n. HDM (*D. pteronyssinus*) every other day for 3 weeks; 3 days later, i.n. of 50 μg of dsRNA every day for 3 days;	BAL granulocytes and lymphocytes Lung tissue alarmins and cytokines Epithelia cell damage Dose-dependent phenotype	Inflammation	C57BL/6 males, 10 week old	(105)
Pneumonia virus of mice (PVM)	i.n. inoculation with 1 PFUs of PVM at 7 days old; reinoculation (20 PFUs) at 42 days post infection (dpi) or with 1 mg cockroach extract at days 3, 45, 52, 59 and 66 dpi. At 94 dpi, the mice were treated with rIL-33 (40 μg/kg) or inoculated with HRV-1B (5.10^6^ TCID50) with or without an IL-33 inhibitor. In some experiments, HDM or LPS was used instead of cockroach extract	Neutrophil, eosinophil and ILC2s infiltration IL-33 and type 2 and 1 cytokine secretion Airway inflammation	Inflammation	BALB/c or 4C13R mice	(106, 107)
Respiratory Syncitial virus (RSV)	i.n. inoculation of 10^5^ PFU; mice harvested 4 days later; also combined with i.n. 1% OVA 11 or 21 dpi: sensitization to 1% OVA by inhalation for 10 consecutive days	Airway hyperresponsiveness Lung eosinophilic and neutrophilic inflammation, Dominance of Th2 cytokines	Inflammation and airway hyperresponsiveness	BALB/c females, 8–12 week old	(108)
Respiratory Syncitial virus (RSV)	i.n. inoculation with 5 × 10^6^ PFUs. Then OVA nebulization for 20 min a day for 10 days. OVA treatment is either at 4–13 dpi, 11–20 dpi or 18–27 dpi	OVA-spec IgG1, OVA-spec IL-4 production in splenocytes OVA-spec IFNg production in CD8 + T cells Association with anaphylaxis at challenge	not reported	BALB/c females, 8–12 week old	(109)
Respiratory Syncitial virus (RSV)	RSV inoculation of 10^4^–10^6^ PFUs. 3 days later, ragweed + Alum inhalation for 5 consecutive days. Or 4 days after infection, mice were i.n. treated with 0.4 mg/ml OVA with or without Alum	Allergen-specific IgE and IgG BAL IgA and IgG	not reported	BALB/c females, 8 to 10 week old	(110, 111)
Influenza A	Pre-sensitization with 20 μg OVA + Alum then at day 7: i.n. infection with Influenza A (−10^4.5^ PFUs). 7 dpi: second sensitization with i.p. OVA/alum. 1 week later, challenge with 1% OVA aerosol or 100 μg HDM i.n.	Increased bronchoalveolar inflammation Eosinophils and neutrophils infiltration Serum OVA-specific IgE Decreased tight junction protein expression in lungs OVA: airway hyperresponsiveness HDM: tissue damping and elastance	Inflammation and airway hyperresponsiveness	BALB/c females, 6 week old	(112)

### Viral infections and allergic asthma

Population studies provide evidence that infections with viral pathogens, such as Human Rhinovirus (HRV) can predispose children to onset of asthma (113, 114). Moreover, once asthma is established, subsequent HRV and Respiratory Syncytial Virus (RSV) infections are most likely culprits causing disease exacerbation (96). In childhood, HRV infections are associated with persistent wheezing, a strong indicator for asthma later in life (115, 116). Many studies linked these virus-mediated exacerbations to increased airway hyperactivity and granulocytic infiltration amongst other factors (117).

Within the last decade, several mouse models have been established to investigate the importance of viral infections for asthma development and exacerbation. We will discuss examples of relevant protocols combining virus and allergen exposures. All cited models are summarized in Table 2.

#### Human rhinovirus

Human rhinovirus (HRV) is the most common causative agent of upper airway tract infections and can exacerbate pre-existing chronic pulmonary disorders (118). Rhinoviruses consist of three phylogenetic species: HRV-A, HRV-B and HRV-C (119). HRV-A and -B rhinoviruses are classified into major or minor group viruses based on their receptor binding and tropism (118, 120). Major group viruses utilize intercellular cell adhesion molecule 1 (ICAM-1) and minor group viruses bind to low-density lipoprotein receptor (LDLR) (118). Major group viruses represent 90% of HRV serotypes and do not bind to murine ICAM-1 (118, 121, 122). For a long time, this represented a roadblock for development of mouse models for this group of rhinovirus infections. This led to the development of a chimeric mouse ICAM-1 which contains the terminal immunoglobulin domain of human ICAM-1 to render mouse cells permissive for major group rhinovirus infections (121–123). In contrast, minor group viruses can bind both human and mouse LDLR (118, 124). Phan and colleagues investigated the effects of infection with a minor group rhinovirus (HRV-1B) following a daily 25 μg dose of HDME for 10 consecutive days (Table 2) (99). The combination of HDME and HRV (as compared to HRV alone) resulted in an exacerbation in total lung leukocytes, particularly neutrophils and macrophages, and a marked increase in total IgE and HDM-specific IgG1. Additionally, hyperresponsiveness to methacholine challenge was significantly increased. This corroborates other studies showing minor group HRVs as an exacerbating factor for OVA-induced asthma (125, 126). Other models relied on high doses of HDME early on followed by multiple lower HDME i.n. administrations and viral infection one day after the last allergen challenge (Table 2) (100–102). These protocols showed a virus-mediated increase in type 1 and type 2 immune responses, including elevated pulmonary eosinophil infiltration, lung type 2 cytokines and airway hyperresponsiveness (100–102). Toussaint and colleagues used a similar HDME sensitization procedure to show that minor group HRV infection exacerbated HDME-induced airway inflammation through the release of host double strand (ds) DNA, in association with neutrophil extracellular traps (NETs) (Table 2) (103). Another approach relied on the use of dsRNA (as an RV mimic) in combination with HDME-induced allergic asthma (105). In this setup, C57BL/6 mice were treated with HDME 3 times a week for 3 weeks to establish asthma, followed by either a low or high i.n. dose of dsRNA on 3 consecutive days then sacrificed 24 h later (Table 2). dsRNA treatment increased the granulocyte and lymphocyte numbers in BAL, as well as lung expression of IL-1β, CCL2 and CCL5 as well as of IL-25, IL-33 and TSLP, a specific group of (predominantly) epithelia-derived cytokines referred to as alarmins. While this study did not specifically test lung function, it reported other lung damage measures such as epithelial shedding and protein exudation.

HRV-C, which was first reported on almost 20 years ago (127, 128), uses the cadherin related family member 3 (CDHR3) as entry receptor on human and mouse cells (129). It has since been found that people with a history of asthma are more susceptible to an HRV-C infection (129–131). To date, this HRV serotype has only been investigated in one study by Rajput et al. in relation to its effect on allergic asthma (Table 2) (104). The infection alone induced a strong type 2 immune response with increased lung mRNA and protein expression of type 2 cytokines as well as expansion of ILC2s, eosinophils, monocytes and neutrophils. In mice pre-treated with HDM before infection with HRV-C15, a similar increase in these cell types, type 2 cytokines and alarmins alongside increased airway hyperresponsiveness was observed. This study provided further relevant insight by comparing the immune responses to HRV-A1B and HRV-C15 infections and found that the latter further increased the airway resistance and eosinophilia in an ILC2-dependent manner (104).

#### Respiratory syncytial virus

In the 1990s, first RSV infection mouse models have shown an exaggerated type 2 immune response after vaccination and natural exposure (132, 133). Through the combination of RSV infection followed by allergen (e.g., ragweed or OVA) sensitization (108–111), these seminal studies increased levels of allergen-specific IgE and IgG as well as BAL IgA and IgG as compared to non-infected mice. Notably, other groups have studied the impact of RSV infection on cockroach extract-mediated sensitization using Incomplete Freund's adjuvant (IFA) during the sensitization phase (134–136). Relying on a similar protocol that includes a systemic (i.p. and s.c.) adjuvant-driven cockroach antigen (CRA) sensitization, followed by RSV infection and CRA challenges, these studies found that RSV indeed exacerbates type 2 immune responses, including eosinophilia and production of cytokines, such as IL-13 and IL-4, as well as airway hyperresponsiveness (134–136). Another recent work used a chronic experimental asthma model combining repeated exposures to pneumonia virus of mice (PVM), a virus closely related to RSV, and cockroach extract for approximately 2 months, followed a month later by the HRV infection [Table 2 (106)]. The infection resulted in a neutrophil-dominated immune response 1 day post infection (d.p.i.), followed by an inflammatory phase between 3 and 7 d.p.i., characterized by increased lung eosinophils and type 2 cytokines as well as mucus hypersecretion. Both phases were abrogated by alarmin depletion and recapitulated by recombinant IL-33 administration. This chronic model, used in other studies, simulates increased airway smooth muscle contraction, a feature of airway remodeling and disease progression observed in humans (107, 137). Combined with the PVM infection, it allowed to identify IL-33 as a main culprit of the observed immune dysregulation. Complex approaches like the one described consider combinations of different stimuli, repeated treatment and resting periods. Therefore, they likely better simulate the exposure processes in humans.

Experimental studies combining RSV and non-adjuvanted allergens such as HDM or pollen extracts could significantly increase our understanding of potential virus-mediated effects on allergic sensitization and asthma.

#### Influenza A virus

Lower respiratory tract influenza A virus (IAV) infections were shown to be associated with asthma exacerbations (138). However, the interplay between asthma and IAV remains more elusive than for RSV and HRV. Addressing the effects of asthmatic state on infection, studies using OVA-based models have shown that chronic asthma can be detrimental for IAV clearance (139) while viral replication is limited in allergic lungs at the early stages of sensitization (140).

On the other hand, only few studies have experimentally investigated the effect of IAV infection on asthma development (109). A recent work using either OVA or HDM as an allergic asthma model showed that influenza pre-infected mice had an increase in lung neutrophils and eosinophils, with a neutrophil-dominated profile for the HDM-treated mice (112). Only the OVA model showed a bronchial hyperresponsiveness phenotype but both allergens induced a decrease in tight junction proteins, such as claudin-1 and occludin (Table 2).

Overall, there is a significant need for more experimental studies investigating the consequences of respiratory viral infections for (chronic) asthma using relevant infection and allergy protocols. Thus far, it remains unclear, for instance, as to whether viral infections are a key trigger of asthma or simply a marker of an inherent predisposition and prior lung dysfunction (141).

### Bacterial infections and allergic asthma

Accumulating evidence suggests that different bacterial species can trigger or exacerbate allergies (142). In the case of asthma, bacteria may aggravate allergic symptoms in conjunction with viruses (HRV or RSV) or alone (96, 143). Since the 1970s, studies documented a correlation between bacterial exposure and allergies. Regarding the common bacterial inhabitants of the human respiratory tract, colonization or infection with *Haemophilus influenzae*, *Streptococcus pneumoniae, Moraxella catharralis* and *Staphylococcus aureus* have been associated with the induction and exacerbation of asthma, chronic obstructive pulmonary diseases and recurrent wheezing early in life (96, 144–150). Moreover, in patients suffering from allergic disorders, such as asthma, atopic dermatitis or nasal polyposis, *S. aureus* colonization appears to occur more frequently (87%, 90%, 87%, respectively), in contrast to 20%–50% colonization of healthy adults (151–153). In addition, asymptomatic colonization of neonates with *S. pneumoniae* or *M. catarrhalis* is associated with later development of recurrent wheezing and asthma (148).

Notably, toxins produced by certain bacteria, such as *Staphylococcus aureus, Bordetella pertussis* and *Streptococcus pneumoniae* have the potential to induce and amplify Th2 immune responses and increase IgE levels in both mice and humans (154–156). An excellent resource by Nordengrün et al. gives an extensive overview on the allergenic potential of bacteria and their overall impact on type 2 immune responses (142). In this section, we will dive into mouse models of bacterial infections that elicit or exacerbate allergic immune responses in the context of asthma. All cited models are summarized in Table 3.

#### Staphylococcus aureus

Several studies have found a correlation between asthma severity and staphylococcal enterotoxin (SE)-specific IgE levels in human patients (110, 144, 157). Experimental work using mouse models has confirmed significant modulation of asthmatic immune responses by skin and airway exposures to *S. aureus* or its toxins. In a work relying on an OVA-induced allergic airway inflammation, i.n. instillations of staphylococcal enterotoxin B (SEB) were found to have a dual role depending on the dose and the timing of the treatment (before or after OVA sensitization) (158). SEB either aggravated or improved different parameters of allergic sensitization and inflammation (Table 3). Another model, using repeated i.t. treatments of the *S. aureus* allergen serine protease-like protein D (SplD), leads to robust, IL-33-dependent asthma in C57BL/6 mice. Interestingly, the potency to induce allergic airway inflammation and asthma features was largely absent in BALB/c mice (159, 160).

**Table 3:** Experimental studies testing the effects of bacterial infections on allergic asthma.

Bacteria	Protocol	Observed allergy features	Presence of airway inflammation and/or hyperresponsiveness	Mice (strain, sex, age)	References
*Staphylococcus aureus* (SEB)	i.n. SEB (50 or 500 ng) on 3 consecutive days either before and/or after weekly i.p. OVA (10 μg) in Alum injections over 3 weeks	Serum IgE Th2 and Th1 responses Granulocyte and lymphocyte infiltration Increased airway resistance	Inflammation and airway hyperresponsiveness	C57BL/6J females, 7–8 weeks old	(158)
*Staphylococcus aureus* (SpID)	6× i.t. SplD (45 μg) every 2 days	IL-33 production BAL eosinophils Mucus hypersecretion SpID-specific IgE	Inflammation	C57BL/6J, C57BL/6N, BALB/c, CBA and DBA/2 females, 6–8 weeks old	(159, 160)
*Staphylococcus aureus* (methicillin resistant)	Depilation with cream and patch application with 10^8^ CFUs of bacteria	IL-17 + eosinophil recruitment Worsened atopic dermatitis	not reported	C57BL/6J females, 8–10 weeks old	(163)
*Staphylococcus aureus* and LTA	shaved, tape-stripped back skin; skin patch containing LTA (25 µg) or 1.1 × 10^9^ CFUs of bacteria; subsequent 3-week patch sensitization with 15 μg HDME for 5 days per week with a change of patch at day 3 and a 2 day rest period, followed by 1× i.n. HDME challenge;	Neutrophil and eosinophil infiltration Type 2 and type 3 cytokines Airway inflammation	Inflammation	BALB/c neonates (7–10 days old) or adult (6 weeks old)	(162)
*Haemophilus influenzae*	i.n 5 × 10^5^ to 1 × 10^6^ CFU of bacteria 3 days after birth old; At 4–5 weeks old, i.p. OVA (16 or 50 µg; + Alum); 1 week later, 15 min high or low OVA dose nebulization for 3 consecutive days;	Immune cell infiltration Lung IL-4 and IL-13; Airway hyperresponsiveness	Inflammation and airway hyperresponsiveness	BALB/c females, 3-day – 6 week old	(165)
*Haemophilus influenzae*	i.p. OVA (50 µg) with Rehydrogel sensitization, ifollowed by i.t. 5 × 10^5^ CFUs of bacteria on the same day or 10 days after or 10 days prior OVA treatment; then days 12–15: i.n. OVA (10 µg) challenge;	Chronic lung inflammation Neutrophil and Th17 cell infiltration Airway hyperresponsiveness Steroid-resistance	Inflammation and airway hyperresponsiveness	BALB/c females, 6–8 weeks old	(166, 167)
*Mycoplasma pneumoniae* (CARDS toxin)	2× i.p. OVA (20 µg + Alum) 2 weeks apart; 2 weeks later: nebulizer challenge with 1% OVA for 20 min for 3 days; 48 h later: i.n. or i.t. of OVA alone or mixed with recombinant CARDS toxin (700 pmol)	Allergic airway inflammation Th2 cytokine production	Inflammation	BALB/c females, 5 weeks old	(168)
*Chlamydophila pneumoniae*	i.n. inoculation with C. pneumoniae with 10^6^ inclusionforming units (IFU) at day 0. Tests were performed at different timepoints following inoculation	Airway hyperresponsiveness and inflammation BAL IFN-*γ* and other cytokines Epithelial damage and secretory cell hypertrophy	Inflammation and airway hyperresponsiveness	BALB/c males, 5–6 weeks old	(169)

Other studies investigated effects of *S. aureus* skin colonization on allergic asthma. The applied models employed either a back skin or ear infection with different strains of the bacterium (161) or exposure to specific bacterial constituents, such as lipoteichoic acid (LTA) (162), serine proteases (163) or toxins (164). *S. aureus* skin infection of mice induces mast cell degranulation, eosinophil recruitment, IgE and IgG1 specific to certain bacteria-derived products, as well as type 2 cytokines (161, 163, 164).

Ubags and colleagues employed an “atopic march” mouse model that that mimics sequential allergen exposure in different body sites by combining dermal and intranasal HDME treatment with or without prior skin pre-exposure to LTA or live *S. aureus* in adult mice (162) (Table 3). This study found that dermal HDME application induced a mixed Th2/Th17 response in BALB/c mice, which was exacerbated by *S. aureus*, with a neutrophil-dominated asthma phenotype. The protocol was also applied in neonates where the *S. aureus*-mediated effects were partially microbiome dependent (162) (Table 3).

While extensive experimental evidence supports the modulatory effect of *S. aureus* on allergic immune responses, the molecular mechanisms driving the interplay between *S. aureus* colonization and asthma exacerbation are still elusive.

#### Pseudomonas aeruginosa

*Pseudomonas aeruginosa* is a ubiquitous bacterium and chronic colonizer of the lower respiratory tract and prevails in patients with chronic airway disorders (170, 171). In asthmatic patients, the presence of *P. aeruginosa* in the sputum is correlated with poorer lung function and increased inflammatory parameters (171). Telford et al. have shown the ability of a *P. aeruginosa*-derived quorum sensing molecule to induce high levels of IgG1 *in vivo* and to stimulate IgE production in IL-4-primed human peripheral blood derived mononuclear cells (172).

#### Haemophilus influenzae

*Haemophilus influenzae* is one of the most commonly identified bacterial pathogens in the airways of patients with severe asthma (173). Several experimental studies used infection mouse models to explore this connection with allergic airway inflammation primarily using mouse models of OVA-induced allergic asthma (Table 3) (165–167, 174). The results demonstrate the potential of *H. influenzae* to influence neutrophil infiltration in asthmatic mice through an IL-17 immune axis (166, 167). In juvenile animals, infection induced a bronchial hyperresponsiveness and increased BAL fluid IL-5 and IL-13 (165). However, we were unable to find experimental studies in context of *H. influenzae* pneumonia using natural allergen (extracts) and omitting adjuvants for sensitization.

#### Atypical bacteria: *Mycoplasma pneumoniae* and *Chlamydophila pneumoniae*

Atypical bacteria, such as *Mycoplasma pneumoniae* and *Chlamydophila pneumoniae*, are challenging to detect by standard methodologies (e.g., Gram stain) (175). In addition, they are difficult to isolate and antibiotic treatment is often ineffective due to their intracellular occurrence (175). *M. pneumoniae* is a common cause of community-acquired pneumonia and can be associated with asthma onset and exacerbations in children and adults (176–179). One experimental study investigated *M. pneumoniae* lung infection in combination with a allergic asthma (168). In this protocol, the mice were sensitized with an i.p. injection of OVA mixed with Alum and challenged 2 weeks later with nebulized OVA for 3 days. This was followed 48 h later by an i.t. or i.n. treatment with OVA and *M. pneumoniae* community-acuired respiratory distress syndrome (CARDS) toxin or with OVA only. Presence of CARDS toxin led to enhanced eosinophilic inflammation and type 2 cytokine production (Table 3).

In a study investigating the impact of *C. pneumoniae* on lung function and inflammation (169), mice were i.n. inoculated with the bacterium, followed by analysis of lung inflammation at different timepoints up to 3 weeks. The infection caused a sustained airway hyperresponsiveness from day 7 to 21, alongside pro-inflammatory cytokine production, expansion of BAL myeloid and lymphoid immune cells and evidence of trachea epithelial cell damage. These results indicate the great potential of *C. pneumoniae* to prime the airways towards higher susceptibility for allergic sensitization or asthma exacerbation.

In summary, further experimental studies with atypical bacteria in context of allergic asthma would be required to better understand this largely unexplored field.

### Fungal infections and allergic asthma

Fungi are ubiquitously present indoors and outdoors and represent one of the largest sources of airborne allergenic substances for humans (180). As such, fungal colonization and exposure to fungal components are considered important factors of asthma development and exacerbations in susceptible individuals (181, 182). Mold allergies are common and can induce a variety of allergic reactions. Several fungal species, such as *Alternaria alternata*, *Aspergiullus fumigatus* or *Cladosporium herbarum*, are potent sensitizing agents in humans (183). Sensitization to some of these fungi is associated with more severe disease, decreased lung function and greater need for corticosteroids for asthma control (184–186). An experimental study investigating the role of chitin, an essential fungal cell wall component (187), has linked this naturally abundant polysaccharide to systemic and pulmonary allergy (188). I.n. chitin instillation of mice led to the lung accumulation of IL-4-producing cells, including eosinophils and basophils. Additionally, this treatment induced alternatively activated macrophages and leukotriene B_4_ production. This important study illustrates the great potential of fungal elements to mobilize immune components usually implicated in allergic responses. In the following section, we will discuss mouse models using fungal infections alone or in combination with allergens in the context of allergic asthma. All models are summarized in Table 4.

#### Alternaria alternata

Mouse models of *Alternaria alternata* infections are relatively well established and generally require either i.p. or i.n. sensitization, followed by repeated i.n. challenges. Asthma features are typically measured one day after the last challenge (Table 4) (189, 190). A study compared the activity of several aeroallergens alone and in combination with *Alternaria* and found that fungal serine protease activity induced IL-33 production in the airways via protease activated receptor (PAR)-2 and adenosine triphosphate signaling. In combination with HDME, *Alternaria* potentiated the pulmonary type 2 inflammation after challenge, resulting in decreased lung function (Table 4) (191). Another related work showed the important role of prostaglandin D_2_ in regulating *Alternaria* extract (AAE)-mediated eosinophil and ILC2 infiltration, as well as pulmonary type 2 immunity (190). Several studies have also reported *Alternaria*-mediated signs of airway remodeling with goblet cell metaplasia and increased airway hyperresponsiveness (Table 4) (189, 192, 193).

**Table 4:** Experimental studies testing the effects of fungal infections on allergic asthma.

Fungi	Models	Observed allergy features	Presence of airway inflammation and/or hyperresponsiveness	Mice (strain, sex, age)	References
*Alternaria alternata* (spores)	i.p. immunization with 2 × 10^6^ spores on days 0 and 7; i.n. challenge with 2 × 10^5^ spores on days 13, 14 and 15;	Eosinophil and neutrophil infiltration Increased airway hyperresponsiveness Goblet cell metaplasia.	Inflammation and airway hyperresponsiveness	BALB/c females, 8–12 week old	(189)
*Alternaria alternata* (extract)	Daily i.n. AAE (5 µg) for 4 consecutive days	Lung eosinophil, ILC2, Th2 cell infiltration; IL-5 and IL-13 Mucus production	Inflammation	BALB/c females, 8–12 week old	(190)
*Alternaria alternata* (extract)	i.n. AAE (50 µg) once or three times (days 0, 3, and 6)	BAL IL-33, IL-5 and IL-13 production and airway eosinophilia without T or B cells	Inflammation	BALB/c males and females, 8–12 week old	(193)
*Alternaria alternata* (extract)	i.n. OVA or ragweed extract (SRW) (both 100 µg) on days 0 and 7, with or without AAE (50 µg); challenge: days 21, 22, and 23 with i.n. OVA or SRW (both 100 µg)	Th2 polarization when using fungal extract with SRW or OVA	Inflammation	BALB/c females, 7–9 week old	(192)
*Alternaria alternata* (extract)	i.n. AAE (10 µg) sensitization, then i.p. (100 µg) and i.n. (12.5 µg) challenge; Exacerbation protocol: i.n. HDM (15 µg) 3×/week for 3 weeks, then one i.n. AAE (10 µg) challenge	Lung IL-33, Th2 cells, esoinophils; Exacerbation protocol: mucus, airway inflammation and hyperresponsivenes, eosinophils, ILC2s, Th2 cells, IL-13	Inflammation and airway hyperresponsiveness	BALB/c females, 6–8 week old	(191)
*Aspergillus fumigatus* (extract and conidia)	Sensitization with extract: s.c. and i.p. AFE (with or without Alum); 2 weeks later: 3× i.n. AFE (20 µg) on consecutive days; challenge with conidia 1 week after last i.n.: i.t. or inhalation (1×, or 2 × 2 weeks after first inhalation or 3 × 4 weeks after second inhalation)	Eosinophil infiltration Serum IgE and IgG Airway remodeling Goblet cell metaplasia Type 2 airway inflammation	Inflammation and airway remodeling	C57BL6/J, CBA/J and BALB/c females, 6–8 week old	(194–196)
*Aspergillus fumigatus* (conidia)	1 × 10^5^ conidia aerosol 2×/week for 4 weeks	BAL macrophages, granulocytes and lymphocytes; Serum IgE and IgG	Inflammation	BALB/c females, 5–7 week old	(197)
*Aspergillus fumigatus*	i.n. treatment with AFE (5 µg) mixed with HDME (5 µg) and ragweed (50 µg) 2×/week for 8 weeks	Chronic asthma with extensive airway remodeling (mucus and collagen deposition) Eosinophil infiltration Airway hyperresponsiveness Type 2 immune response and serum IgE and IgG	Inflammation and airway hyperresponsiveness	BALB/c females, 12–15 week old	(198)
*Aspergillus oryzae* (protease)	i.n. treatment with 90 µg of protease ×4/week for 2 weeks	Eosinophil infiltration Airway resistance	Inflammation and airway hyperresponsiveness	C57BL/6 and BALB/c mice	(199)
*Cladosporium herbarum*	i.p. immunization with 2 × 10^6^ spores on days 0 and 7; i.n. challenge with 2 × 10^5^ spores on days 13, 14 and 15	Eosinophil and neutrophil infiltration IgE, IgG1 and IgM	Inflammation	BALB/c females, 8–12 week old	(189)

#### Aspergillus

*Aspergillus fumigatus* is a fungal pathogen that can be found in water, soil, food and decomposing vegetation (200). In its conidial (spore) form, *A. fumigatus* can be readily airborne and inhaled (200). Initially, *A. fumigatus* infection mouse models used cultured fungus until conidia-based models emerged and were shown to recreate the immunopathological features of fungal asthma (180). The routes of delivery vary between models for sensitization and challenge phases. A study comparing the asthma phenotype between i.t. and inhalation routes of conidia delivery during challenge found that inhalation results in a more enhanced eosinophilic inflammation, serum IgE and airway remodeling (194). Similar models with inhalation challenge showed sustained airway remodeling and type 2 inflammation (195, 196). Importantly, the choice of live vs. irradiated (dead) conidia plays a major role in the character and intensity of the allergic immune response as live conidia elicit more robust inflammation and airway remodeling features in fungal asthma (201).

Another study characterized the lung immune responses to repeated inhalation of conidia aerosolized using an acoustical generator. This approach mimics the exposure to fungal bioaerosols that are typically encountered in the environment (197). This protocol led to increased BAL macrophages, granulocytes, and lymphocytes as well as specific IgG.

Goplen and colleagues established a chronic asthma model utilizing a mixture of one, two or three allergens derived from HDM, ragweed and *Aspergillus* without adjuvant (198). I.n. exposure to the triple allergen mix twice a week for 8 weeks resulted in chronic airway inflammation accompanied by impaired lung function, a profound type 2 immune response and *Aspergillus*-specific IgE and IgG production.

*Aspergillus oryzae* is a fungus of significant economic importance as its α-amylase enzyme is broadly used in the pharmaceutical and food industry. Purified *A. oryzae* protease is a potent allergen (199) as its i.n. application (4 times a week for 2 weeks) induces robust BAL eosinophilic infiltration and increased airway resistance. This study highlights the great allergenic potential of exogenous proteases upon airway exposure.

#### Cladosporium herbarum

*Cladosporium herbarum* is a common fungus of global clinical relevance (202). Alongside *Alternaria*, Havaux et al. tested the allergenicity of *C. herbarum* spores (189) and found that two i.p. immunizations with a 1 month interval resulted in significantly increased IgE levels 15 days later. I.n. challenge of sensitized animals led to increased BAL type 2 cytokines (IL-4, IL-5 and IL-13) as well as airway hyperresponsiveness.

Overall, fungi and fungal components are amongst the most potent and important environmental triggers of allergic asthma. However, due to the scarcity of experimental studies, significant questions related to immunopathogenesis and consequences of fungal exposures remain unanswered.

## Conclusion and future perspectives

The complexity of pathological immune responses in asthma requires elaborate animal models that consider and reflect this intricacy. In addition, this disease comprises a variety of endotypes and severity grades. The development of protocols that incorporate clinically relevant exposure routes and allergens has brought a significant benefit to this field of research. Furthermore, models of chronic disease are of great importance and have emerged as an indispensable tool to replicate the pathophysiology of asthma and identify the long-term consequences of allergen exposure and repeated exacerbations. However, a significant type of airway allergy that still lacks an adjuvant-free mouse model (independent of chemical adjuvants or systemic immunization) is pollen allergy. Additionally, incorporating a broader range of aeroallergens or utilizing combinations of different allergens would provide a more comprehensive understanding of the complex implications of environmental exposures as observed in humans.

Finally, further research investigating concomitant airway infections and asthma could guide towards new preventative and therapeutic strategies. For instance, potential associations between disease features or clinical biomarkers and pathogen group signatures in mice and humans could address some aspects of treatment resistance and have a great impact on disease management and outcome.
